# Elevated Expression of C-Type Lectin Domain Family 5-Member A (CLEC5A) and Its Relation to Inflammatory Parameters and Disease Course in Adult-Onset Still's Disease

**DOI:** 10.1155/2020/9473497

**Published:** 2020-04-23

**Authors:** Po-Ku Chen, Shie-Liang Hsieh, Joung-Liang Lan, Chi-Chen Lin, Shih-Hsin Chang, Der-Yuan Chen

**Affiliations:** ^1^Translational Medicine Laboratory, Rheumatology and Immunology Center, China Medical University Hospital, Taichung, Taiwan; ^2^Rheumatology and Immunology Center, China Medical University Hospital, Taichung, Taiwan; ^3^College of Medicine, China Medical University, Taichung, Taiwan; ^4^Institute of Clinical Medicine, National Yang-Ming University, Taipei, Taiwan; ^5^Genomics Research Center, Academia Sinica, Taipei, Taiwan; ^6^Rheumatic Diseases Research Center, China Medical University Hospital, Taichung, Taiwan; ^7^Ph.D. Program in Translational Medicine and Rong Hsing Research Center for Translational Medicine, National Chung Hsing University, Taichung, Taiwan; ^8^Institute of Biomedical Science, National Chung Hsing University, Taichung, Taiwan

## Abstract

C-type lectin domain family 5-member A (CLEC5A) associates with adaptor DAP12 (DNAX activation protein 12) to form receptor complexes involved in inflammatory responses. We postulated a potential role of CLEC5A in the pathogenesis of adult-onset Still's disease (AOSD) and aimed to investigate CLEC5A expression and its association with activity parameters and disease course. In 34 AOSD patients and 12 healthy controls (HC), circulating levels of CLEC5A-expressing monocytes or granulocytes were determined by flow cytometry analysis, the mRNA expression of CLEC5A and DAP12 on PBMCs by quantitative PCR, and plasma levels of proinflammatory cytokines by ELISA. AOSD patients had significantly higher percentages and mean fluorescence intensity (MFI) of CLEC5A-expressing monocytes (median 62.1% and 3.20, respectively) or granulocytes (72.6% and 3.22, respectively) compared with HC (in monocytes: 17.0% and 0.65, both *p* < 0.001; in granulocytes: 67.3%, *p* < 0.05 and 0.90, *p* < 0.001; respectively). Patients also had significantly higher levels of CLEC5A mRNA expression on PBMCs compared with HC (median 1.77 vs. 0.68, *p* < 0.05). The levels of CLEC5A-expressing monocytes or granulocytes were positively associated with activity scores and levels of IL-1*β* and IL-18 in AOSD patients. The patients with a systemic pattern had significantly higher levels of CLEC5A-expressing granulocytes and IL-18 compared to those with a chronic articular pattern of disease course. After 6 months of therapy, levels of CLEC5A-expressing monocytes and granulocytes significantly declined, paralleling the decrease of AOSD activity. Elevated CLEC5A levels and their positive association with activity parameters suggest that CLEC5A is involved in the pathogenesis and may serve as an activity indicator of AOSD.

## 1. Introduction

Adult-onset Still's disease (AOSD) is characterized by spiking fever, skin rash, arthritis, multisystemic involvement, neutrophilic leukocytosis, and elevated levels of acute phase reactants [1–3], and the affected tissues, such as skin, show influx of neutrophils [4]. AOSD has recently been considered an autoinflammatory disease (AID) due to its characteristic phenotypes and the absence of detectable autoantibodies [5]. Accumulating evidence indicates that dysregulated inflammasome plays a pathogenic role in AID [6, 7]. In AOSD patients, the rapid response to IL- (interleukin-) 1*β* inhibitors [8] suggests the critical role of IL-1*β*-related inflammasome in its pathogenesis. Resonating with this finding, we recently revealed an elevated expression of NLRP3-inflammasome signaling in AOSD patients [9].

The C-type lectin domain family 5-member A (CLEC5A) or myeloid DAP12-associated lectin-1 (MDL-1) associates noncovalently with adaptor DAP12 (DNAX activation protein 12) to form receptor complexes which are involved in inflammatory responses [10, 11]. Our previous study revealed elevated CLEC5A expression on peripheral mononuclear cells (PBMCs) and inflamed synovium in patients with rheumatoid arthritis (RA) [12]. Aoki et al. also reported that mouse macrophages and neutrophils had significantly increased expression of CLEC5A, which played an important role in innate immunity [11]. In addition, previous studies demonstrated that the dengue virus could activate NLRP3-inflammasome through CLEC5A [13], and the blockade of CLEC5A could inhibit inflammasome activation and abolish dengue virus-induced IL-1*β* production [13, 14]. Given the neutrophilic leukocytosis and elevated expression of NLRP3-inflammasome characteristic of AOSD [9], we hypothesize an important role of CLEC5A in AOSD pathogenesis.

In this pilot study, we investigated the differences in (i) the CLEC5A expression levels in circulating monocytes and granulocytes and (ii) the mRNA expression levels of CLEC5A and DAP12 between AOSD patients and healthy control (HC) individuals. The correlation between CLEC5A levels and disease activity scores or inflammatory parameters in AOSD patients was also evaluated. In addition, we examined the association between CLEC5A levels and disease outcome in AOSD patients.

## 2. Materials and Methods

### 2.1. Subjects

Thirty-four active AOSD patients were enrolled in this study, each fulfilling the Yamaguchi criteria [15]. Patients with infections, malignancies, or other rheumatic diseases were excluded. The disease activity of each AOSD patient was assessed by a modified Pouchot score [16], and active AOSD was defined as an activity score of at least 3. All patients had received therapy with corticosteroids and/or the nonsteroidal anti-inflammatory drugs at an active status. Besides, they have received at least one of the disease-modifying antirheumatic drug (DMARD) therapies including methotrexate (*n* = 26), hydroxychloroquine (*n* = 22), azathioprine (*n* = 6), and cyclosporine (*n* = 5). Defined as described in previous studies [17, 18], AOSD patients who had been followed for at least one year were classified into two patterns of disease course: a systemic pattern that includes the monocyclic and polycyclic forms and the other a chronic articular pattern (persistent arthritis involving at least one joint and lasting longer than 6 months). Twelve healthy volunteers, who did not have any rheumatic disease, were enrolled as control subjects. The present study was approved by the Institutional Review Board of our hospital (CMUH107-REC3-094), and each participant's written consent was obtained according to the Declaration of Helsinki.

### 2.2. Quantitation of CLEC5A-Expressing Cells Using Flow Cytometry Analysis

To quantify CLEC5A expression levels in granulates and monocytes, 1 ml samples of whole blood were collected and stained with phycoerythrin- (PE-) conjugated anti-CLEC5A monoclonal antibody (mAb) (R&D Systems, Minneapolis, MN, USA) and phycoerythrin-cyanin 5- (PC5-) conjugated anti-CD14 mAb (Beckman Coulter, Brea, CA, USA) or fluorescein isothiocyanate- (FITC-) conjugated CD66b-specific mAb (Beckman Coulter, Brea, CA, USA) according to the manufacturer's protocol and the described technique [12]. Mouse IgG2b-PE (R&D Systems, Minneapolis, MN, USA) and IgG2a-PC5 (Beckman Coulter, Brea, CA, USA) were used as isotype controls. Samples were incubated with antibodies for 20 minutes in the dark at room temperature, and then, erythrocytes were lysed by 500 *μ*l of OptiLyse C Lysis Solution (Beckman Coulter, Brea, CA, USA) for 10 minutes to lyse red blood cells. Then, the cells were resuspended with 500 *μ*l PBS/each tube prior to flow cytometry (Beckman Coulter, Brea, CA, USA) analysis. Monocytes and granulocytes were gated based on CD45+/side scatters (SSC) and at least 2 × 10^5^ total cells from each sample were analyzed. Data were expressed as the percentages or the mean fluorescence intensity (MFI) of CLEC5A expression in circulating monocytes or granulocytes.

### 2.3. Quantitative PCR Analysis for mRNA Expression of CLEC5A and DAP12 on PBMCs

Given that human CLEC5A mRNA is not expressed in granulocytes [10], we examined transcript levels only in PBMCs. The PBMCs (17 AOSD patients and 9 healthy subjects) were immediately isolated using Ficoll-Paque™ Plus (GE Healthcare Bio-Sciences AB, Uppsala, Sweden) density gradient centrifugation. Total RNA from PBMCs was isolated by the guanidinium isothiocyanate method [19]. A RNA (2.5 *μ*g) aliquot was reverse transcribed with reverse transcriptase of Moloney murine leukemia virus (Fermentas, Thermo Fisher Scientific Inc., MD, USA). The qPCR was performed using the TOOLS 2x SYBR qPCR Mix (Biotools Co., New Taipei, Taiwan) as described in previous reports [20]. Sequences for designing the primers in this study are listed below: CLEC5A, sense primer 5′-GTAACGATGGTTTCACCACC-3′ and antisense primer 5′-GCCACCTTTTCTCTTCACGA-3′; DAP12, sense primer 5′-GGACTTGAACCCTGCAGCAG-3′ and antisense primer 5′-TACGCTGTTTCCGGGTCGCT-3′; and the housekeeping gene GAPDH, sense primer 5′-GAAGGTGAAGGTCGGAGTC-3′ and antisense primer 5′-GAAGATGGTGATGGGATTTC-3′. Quantitative PCR (a total volume of 20 *μ*l) was conducted using cDNA (10 ng), TOOLS 2x SYBR qPCR Mix (10 *μ*l), each oligonucleotide primer (0.6 *μ*l), and RNase-free water. The cycling conditions have been described previously [21]. The mRNA expression levels of CLEC5A and DAP12 were normalized to the control gene GAPDH. The relative expression levels of CLEC5A and DAP12 were calculated using the comparative threshold cycle (Ct) method and evaluated using 2^−*∆∆*Ct^ as described previously [21].

### 2.4. The Effects of CLEC5A Knockdown on NLRP3-Inflammasome Expression on THP-1 Cell

Human monocytic THP-1 cells (BCRC 60430, the Bioresource Collection and Research Center, Taiwan) were grown in RPMI 1640 (Gibco, Thermo Fisher Scientific Inc.) with glutamine (Invitrogen, Carlsbad, CA, USA) medium supplemented with 10% fetal bovine serum in an incubator containing 5% CO_2_ at 37°C. THP-1 cells were differentiated into macrophage-like cells by incubation with 10 nM PMA (phorbol 12-myristate 13-acetate) (Sigma-Aldrich, St. Louis, MO, USA) for 48 hours. To examine the effect of CLEC5A in NLRP3-inflammasome expression by using an in vitro cell-based assay, the cells were transiently transfected with CLEC5A siRNA (cat# D-001810-10-05, Dharmacon, Lafayette, CO, USA) or control siRNA (cat# D-001810-10-05, Dharmacon, Lafayette, CO, USA) using the DharmaFECT Transfection Reagent (Qiagen, Valencia, CA, USA) for 48 hours. To confirm the efficacy of transfection, cells were harvested for subsequent detection of CLEC5A expression by Western blotting. For inducing the activation of NLRP3-inflammasome, the siCLEC5A knockdown THP-1 cells were treated with plasma from active AOSD patients or healthy controls for 6 hours. NLRP3-inflammasome expression levels from cell lysates were then analyzed by Western blotting.

### 2.5. Western Blotting for NLRP3-Inflammasome Expression Levels

Total proteins were extracted from lysates of THP-1 cells treated with plasma from active AOSD patients or healthy controls. The samples were run on 10% SDS-PAGE and then transferred to PVDF membranes (Bio-Rad, Hercules, CA, USA). The blots were blocked with 5% milk in PBS with 0.1% Tween-20 (PBST) (Bionovas, Inc., Washington, DC, USA) for 30 min at room temperature, and subsequently incubated with specific anti-CLEC5A antibody (Aviva Systems Biology, San Diego, CA, USA), anti-NLRP3 antibody (Cell Signaling Technology, Beverly, MA, USA), anti-caspase-1 antibody (Abcam, Cambridge, MA, USA), anti-IL-1*β* antibody (Novus Biologicals, LLC, Littleton, CO, USA), anti-IL-18 antibody (Medical & Biology Laboratories Co, Ltd., Naka-ku, Nagoya, Japan), and anti-*α*-tubulin (1: 5000, Santa Cruz Biotechnology, Dallas, Texas, USA) at 4°C overnight. After washing with PBST, the membranes were incubated with horseradish peroxidase-conjugated secondary antibody (Thermo Fisher Scientific Inc., Pittsburgh, PA, USA). Immunoreactive bands were incubated with an ECL detection system (Advansta, Menlo Park, CA, USA) and visualized by radiographic film. The band intensity was quantitated by ImageJ software as described previously [22]. The protein levels of NLRP3, caspase-1, IL-1*β*, and IL-18 were normalized to *α*-tubulin.

### 2.6. Determination of Levels of IL-1*β* and IL-18 Using ELISA

Plasma levels of proinflammatory cytokines were measured by ELISAs, including IL-1*β* (RayBiotech Inc., Norcross, GA, USA) and IL-18 (Medical & Biology Laboratories Co, Ltd., Naka-ku, Nagoya, Japan) based on each of the manufacturer's instructions and as described previously [22]. All assays were determined with both interassay and intra-assay coefficient of variation (CV) of less than 10%.

### 2.7. Statistical Analysis

Results are presented as the mean ± standard deviation (SD) or median (interquartile range). The nonparametric Kruskal-Wallis test was used for comparisons between groups. When this test showed a significant difference, the exact *p* value was determined using the Mann-Whitney *U* test. The correlation coefficient was calculated using the nonparametric Spearman's rank correlation test. The Wilcoxon signed rank test was employed to compare the expression levels of both NLRP3-inflammasome and downstream cytokines before and after treatment. A *p* value < 0.05 was considered statistically significant.

## 3. Results

### 3.1. Demographic Data and Clinical Characteristics of AOSD Patients

Among the 34 AOSD patients (age at study entry: mean ± SD, 42.3 ± 12.3 years; 24 women and 10 men) enrolled in this study, the presence of fever (≥39°C), skin rash, sore throat, arthralgia or arthritis, liver dysfunction, and lymphadenopathy were observed in 33 (97.1%), 30 (88.2%), 26 (76.5%), 22 (64.7%), 16 (47.1%), and 11 (32.4%), respectively. However, there were no significant differences in the demographic data between AOSD patients and healthy controls (44.8 ± 9.8 years; 9 women and 3 men).

### 3.2. The Percentages and MFI of CLEC5A-Expressing Monocytes and Granulocytes

**Figure 1:**
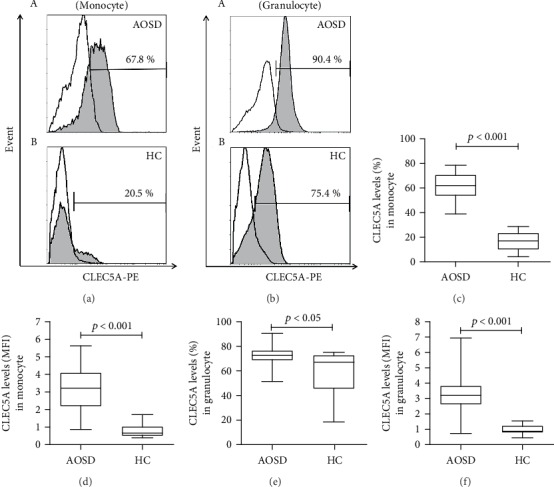
The percentages and MFI of CLEC5A-expressing monocytes and granulocytes in patients with adult-onset Still's disease (AOSD). Representative examples of flow cytometric histograms of CLEC5A-expressing monocytes or granulocytes obtained from peripheral blood of one active AOSD patient ((a) A) or ((b) A) and one healthy subject ((a) B) or ((b) B). Comparisons of the percentages and mean fluorescence intensity (MFI) of CLEC5A-expressing monocytes ((c) and (d)) or granulocytes ((e) and (f)) between AOSD patients and healthy control subjects. The data are presented as box-plot diagrams, in which the box encompasses the 25th percentile (lower bar) to the 75th percentile (upper bar). The horizontal line within the box indicates the median value for each group. The *p* values were determined by using the nonparametric Mann-Whitney *U* test.

The representative cytometric histograms of CLEC5A expression on monocytes or granulocytes were obtained from one active AOSD patient (Figure 1(a) A and B) and one healthy subject (Figure 1(b) A and B), respectively. As shown in Figures 1(c) and 1(d), significantly higher percentages and MFI of CLEC5A-expressing monocytes were observed in AOSD patients (median 62.1%, interquartile range (IQR) 53.4-70.8%; 3.20, IQR 2.15-4.10; respectively) compared with those in HC (17.0%, IQR 10.0-23.7%; 0.65, IQR 0.49-1.03; respectively, both *p* < 0.001, Figures 1(c) and 1(d)). Similarly, significantly higher percentages and MFI of CLEC5A-expressing granulocytes were observed in AOSD patients (median 72.6%, IQR 68.3-76.5%; 3.22, IQR 2.60-3.81; respectively) compared with those from HC (67.3%, IQR 45.5-72.7%, *p* < 0.05; 0.90, IQR 0.76-1.25, *p* < 0.001; respectively, Figures 1(e) and 1(f)).

### 3.3. The mRNA Expression Levels of CLEC5A and DAP12

**Figure 2:**
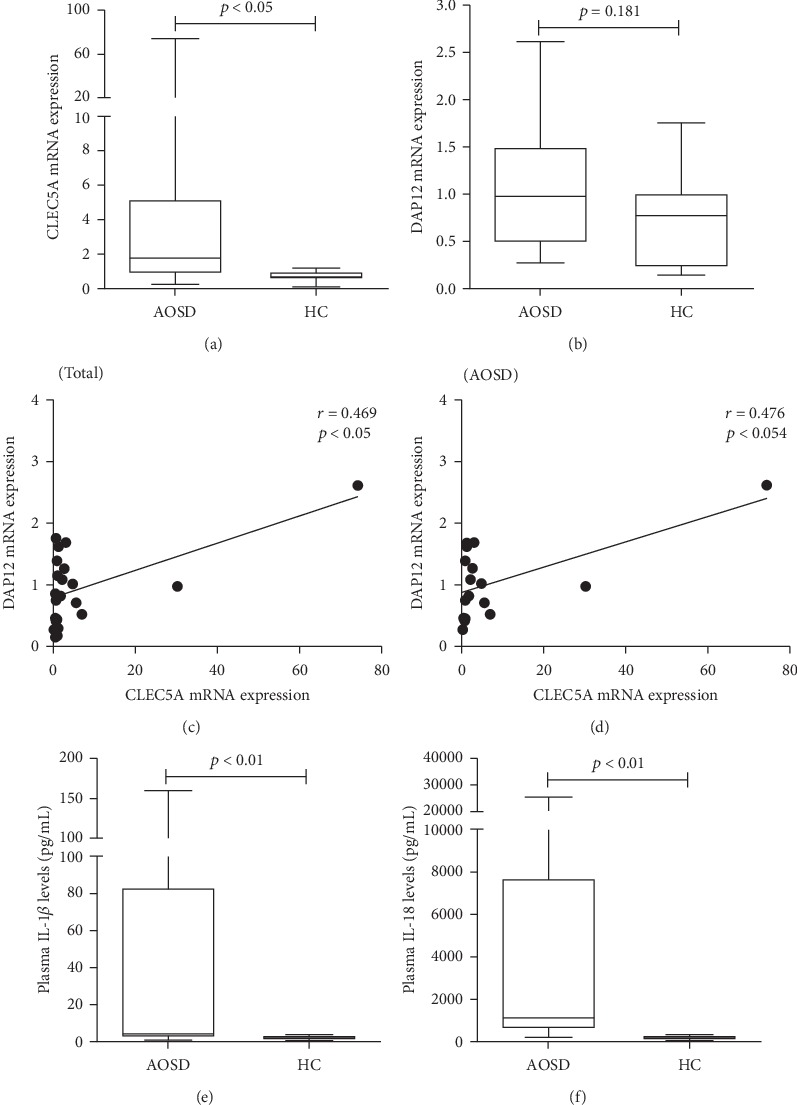
The mRNA expression levels of CLEC5A and DAP12 and the level of inflammasome-related cytokines IL-1*β* and IL-18 in patients with AOSD. The comparisons of relative mRNA expression levels of CLEC5A and DAP12 on PBMCs ((a) and (b), respectively) between 17 AOSD patients and 9 healthy subjects. The horizontal line indicates the median value for each group. The correlation between CLEC5A expression and DAP12 expression in all subjects (c) or AOSD patients (d). Correlation coefficients (*γ*) and *p* value were obtained by the nonparametric Spearman's rank correlation test. The comparisons in plasma levels of proinflammatory cytokines including IL-1*β* (e) and IL-18 (f) from active AOSD patients and healthy controls (HC). The data are presented as box-plot diagrams, in which the box encompasses the 25th percentile (lower bar) to the 75th percentile (upper bar). The horizontal line within the box indicates the median value for each group. The *p* values were determined by using the nonparametric Mann-Whitney *U* test. CLEC5A: C-type lectin domain family 5-member A; DAP12: DNAX activation protein 12; AOSD: adult-onset Still's disease; PBMCs: peripheral blood mononuclear cells.

As shown in Figure 2(a), AOSD patients had significantly higher levels of CLEC5A mRNA expression on PBMC (median 1.77, IQR 0.84-5.19) compared with HC (0.68, IQR 0.53-1.00, *p* < 0.05). DAP12 mRNA expression levels were also higher in AOSD patients (0.98, IQR 0.49-1.51) than in HC (0.77, IQR 0.23-1.01, Figure 2(b)), although it did not reach statistical significance (*p* = 0.181). As illustrated in Figures 2(c) and 2(d), CLEC5A mRNA expression levels were positively correlated with DAP12 expression levels on PBMCs from AOSD patients (correlation coefficient *R* = 0.475, *p* = 0.054) and from all subjects (*R* = 0.469, *p* < 0.05).

### 3.4. Plasma Levels of IL-1*β* and IL-18

As shown in Figures 2(e) and 2(f), significantly higher levels of IL-1*β* and IL-18 were observed in AOSD patients (median 5.0 pg/ml, IQR 2.2-83.5 pg/ml; 1088 pg/ml, IQR 590-7719 pg/ml; respectively) than in HC (median 2.1 pg/ml, IQR 1.4-3.1 pg/ml, *p* < 0.05; 109 pg/ml, IQR 70-152 pg/ml, *p* < 0.001; respectively).

### 3.5. Correlations of CLEC5A Expression with Inflammatory Parameters in AOSD Patients

As illustrated in Table 1, the frequencies of CLEC5A in circulating monocytes and granulocytes were positively associated with systemic activity scores and serum ferritin levels in AOSD patients. The frequencies of CLEC5A-expressing monocytes were also positively associated with plasma IL-1*β* levels, and the MFI of CLEC5A-expressing granulocytes were positively associated with plasma IL-18 levels in AOSD patients.

### 3.6. The Difference in the Effects of CLEC5A Knockdown on NLRP3-Inflammasome Expression in THP-1 Cells between AOSD Patients and Healthy Controls

**Figure 3:**
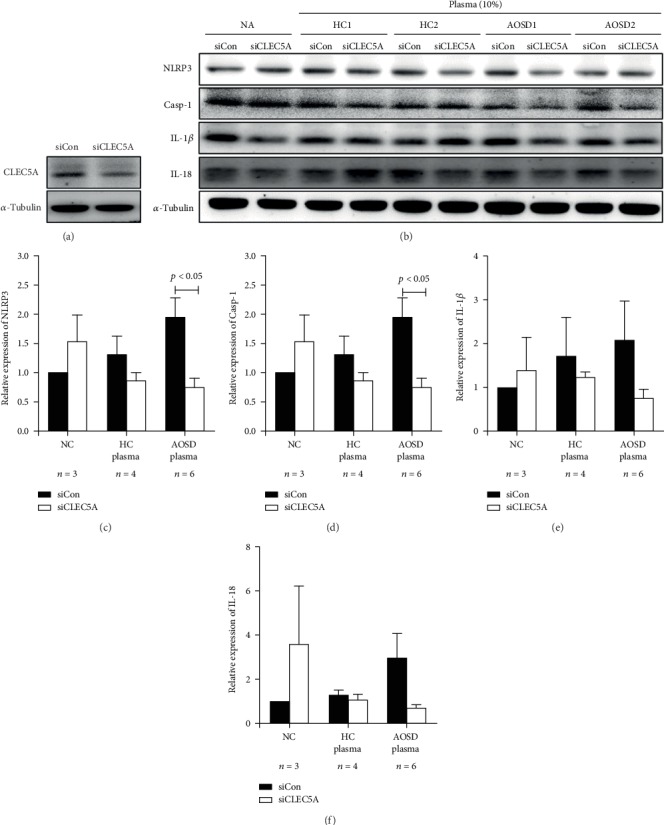
The difference in the effects of CLEC5A knockdown on NLRP3-inflammasome expression in THP-1 cells between AOSD patients and healthy controls. (a) CLEC5A expression in THP-1 cells was knocked down by CLEC5A-specific siRNA (Dharmafect) and a nonspecific siRNA (negative control) for 48 hours. (b) Cells were stimulated with 10% plasma from AOSD patients (*n* = 6) and HC (*n* = 4) for 6 h at 37°C, and cell lysates were harvested for the determination of (c) NLRP3, (d) caspase-1, (e) IL-1*β*, and (f) IL-18 by Western blotting. *α*-Tubulin is used as loading control. AOSD: adult-onset Still's disease; HC: healthy controls; IL: interleukin. Bars and error bars indicate mean and standard deviation, respectively. The *p* values were determined by Bonferroni's posttest.

At first, we revealed a downregulation of CLEC5A expression in THP-1 cells transfected with siCLEC5A (Figure 3(a)). Among THP-1 cells without CLECD5A knockdown (siCon), there was a trend of increased expression of NLRP3-inflammasome signaling in AOSD patients compared with healthy controls (HC) (Figures 3(b)–3(f)). On the other hand, the protein expression levels of NLRP3 and caspase-1 could be significantly suppressed in THP-1 cells knocked down with siCLEC5A (fold change: 1.96 ± 0.79 versus 0.76 ± 0.33, *p* = 0.019; 1.98 ± 0.078 versus 0.76 ± 0.33, *p* = 0.020; respectively) (Figures 3(b)–3(f)).

### 3.7. The Differences in CLEC5A Levels and Plasma Cytokine Levels in AOSD Patients with Different Patterns of Disease Course

**Figure 4:**
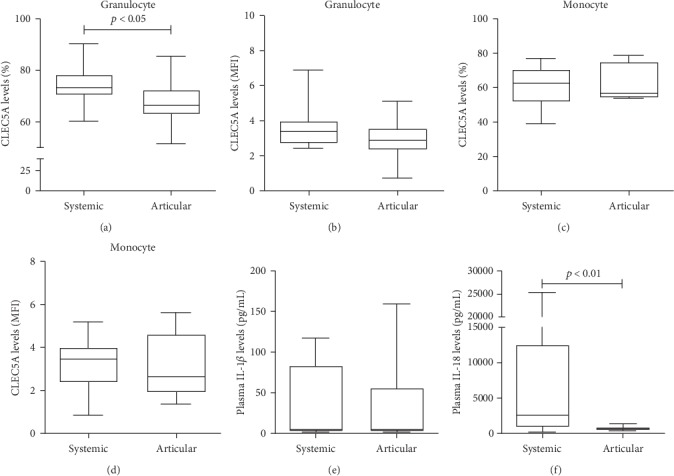
The differences in CLEC5A levels and plasma cytokine levels in AOSD patients with different patterns of disease course. The differences in the percentages and MFI of CLEC5A-expressing granulocytes ((a) and (b), respectively) or monocytes ((c) and (d), respectively), and plasma levels of IL-1*β* (e) and IL-18 (f) in AOSD patients with different patterns of disease course. Data are presented as box-plot diagrams, with the box encompassing the 25th percentile (lower bar) to the 75th percentile (upper bar). The horizontal line within the box indicates median value, respectively, for each group. The *p* values were determined by the Mann-Whitney *U* test.

Among the 34 AOSD patients, 25 (73.5%) had a systemic pattern and the remaining 9 (26.5%) had a chronic articular pattern. As shown in Figure 4, AOSD patients with a systemic pattern had significantly higher frequencies of CLEC5A in granulocytes and plasma IL-18 levels (median 73.3%, IQR 70.4-78.2%; 2554 pg/ml, IQR 815-12554 pg/ml; respectively) compared with those with a chronic articular pattern (66.4%, IQR 62.9-72.5%, *p* < 0.05; 622 pg/ml, IQR 441-940 pg/ml, *p* < 0.01; respectively). However, there was no significant difference in CLEC5A levels in monocytes or plasma IL-1*β* levels between patients with different patterns.

### 3.8. Changes in CLEC5A Levels in Immune Cells from AOSD Patients after Therapy

**Figure 5:**
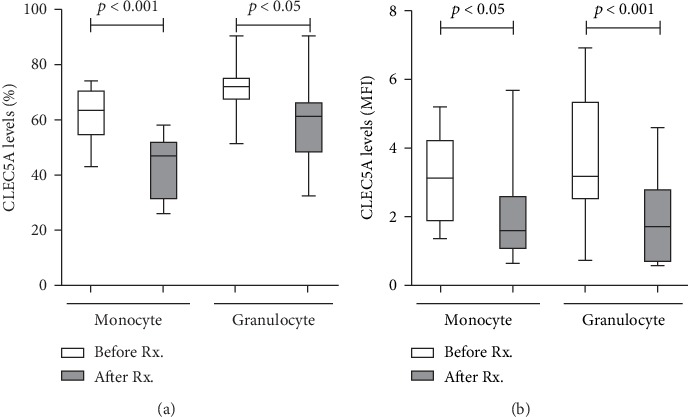
The change of CLEC5A levels on immune cells from AOSD patients after therapy. The changes of (a) the frequencies or (b) the mean fluorescence index (MFI) of CLEC5A-expressing monocytes and granulocytes in AOSD patients after 6-month therapy. Data are presented as box-plot diagrams, with the box encompassing the 25th percentile (lower bar) to the 75th percentile (upper bar). The horizontal line within the box indicates median value, respectively, for each group. The *p* values were determined by the Wilcoxon signed rank test.

Twelve active AOSD patients were available for examination of their CLEC5A levels at the active phase (as baseline) and at week 24 of therapy. As shown in Figure 5, the percentages and MFI of CLEC5A-expressing monocytes significantly declined after therapy (median 63.5%, IQR 54.1-71.3% versus 47.0%, IQR 31.0-52.4%, *p* < 0.005; 3.12, IQR 1.84-4.27 versus 1.62, IQR 1.03-2.63, *p* < 0.05; respectively), paralleling the decreases of AOSD activity score (5.00, IQR 4.00-5.75 versus 2.00, IQR 1.00-2.75, *p* < 0.005). Similarly, the percentages and MFI of CLEC5A-expressing granulocytes significantly declined (median 72.1%, IQR 66.9-75.6% versus 61.4%, IQR 47.8-66.7%, *p* < 0.05; 3.19, IQR 2.48-5.38 versus 1.73, IQR 0.67-2.84, *p* < 0.01; respectively) along with the decreases in activity score.

## 4. Discussion

The implication of CLEC5A in innate immunity has been established due to its role in the differentiation and activation of monocytes and neutrophils [10, 11]. However, the relation between CLEC5A expression and AOSD pathogenesis remains unexplored. The present study is the first to demonstrate significantly higher levels of CLEC5A in circulating monocytes and granulocytes from AOSD patients compared with those from healthy subjects. The CLEC5A mRNA expression levels were significantly elevated as well. Moreover, CLEC5A levels in circulating monocytes and granulocytes were positively associated with AOSD activity score and inflammasome downstream cytokines such as IL-1*β* and IL-18. We also demonstrated that CLEC5A knockdown could significantly suppress the expression of NLRP3-inflammasome expression. During the longitudinal follow-ups, disease remission in AOSD patients was accompanied by a paralleling, significant decrease of CLEC5A expression levels. Besides, AOSD patients with a systemic pattern of disease course had significantly higher frequencies of CLEC5A in granulocytes and plasma IL-18 levels compared to those with a chronic articular pattern. These observations suggest that CLEC5A level is indicative of disease activity and related to disease course in AOSD.

CLEC5A, a type II transmembrane protein belonging to the C-type lectin family, is expressed on monocytes [10], and its expression is notably induced on monocyte or neutrophil differentiation [11, 23]. The significant elevation of CLEC5A levels in circulating monocytes and granulocytes from our AOSD patients is in accordance with other previous findings of increased CLEC5A expression on murine bone marrow macrophages [10] and induction of CLEC5A expression upon neutrophil activation [11, 23]. The elevated CLEC5A levels not only were positively associated with disease activity scores and serum ferritin levels but also deceased in parallel with disease remission in AOSD patients. These observations suggest that CLEC5A overexpression may play an important role in AOSD pathogenesis. However, further larger prospective studies should be conducted to confirm these findings.

To verify the increased CLEC5A expression at transcript levels in AOSD patients, qPCR for CLEC5A mRNA expression were performed on PBMCs from our active AOSD patients. We demonstrated that the relative expression levels of CLEC5A mRNA were significantly higher in AOSD patients than in HC. Because CLEC5A lacks signaling motifs, it requires an association with adaptor DAP12 to generate the signaling pathway [11]. In the present study, there was a positive correlation between CLEC5A expression levels and DAP12 levels in AOSD patients and all subjects. Such results support the findings that CLEC5A expression is noncovalently coupled with adaptor DAP12 to form CLEC5A receptor complexes, which are involved in the inflammatory responses [11, 24].

As expected in a systemic inflammatory disease, plasma levels of proinflammatory cytokines, including NLRP3-inflammasome downstream cytokines IL-1*β* and IL-18, were significantly increased in active AOSD patients compared with HC. In agreement with previous finding that CLEC5A crosslinking could trigger the secretion of proinflammatory cytokines [10, 14, 24, 25], plasma levels of IL-1*β* and IL-18 were positively associated with CLEC5A levels in monocytes or granulocytes from our AOSD patients. Given the involvement of IL-1*β* and IL-18 in the inflammatory response in AOSD [9, 26–28], CLEC5A seems to be an amplifier of the innate immune response.

The blockade of CLEC5A has been shown to inhibit inflammasome activation and abolish dengue virus-induced IL-1*β* production [13, 14]. Our result showed that CLEC5A knockdown could significantly suppress the protein expressions of NLRP3 and capase-1 in THP-1 cells treated with plasma from AOSD patients compared to those from HC. Although there was no statistical significance, the expression level of IL-1*β* and IL-18 could also be suppressed by CLEC5A knockdown cells. These findings suggest a role of CLEC5A in NLRP3-inflammasome-mediated inflammation in AOSD patients, although the exact mechanism remains to be elucidated.

Disease course of AOSD patients may vary considerably [17, 18], and none of the clinical or laboratory variables could reliably predict the disease course. We revealed that patients with a systemic pattern had significantly higher frequencies of CLEC5A in granulocytes and higher IL-18 levels than those with a chronic articular pattern, which is consistent with previous findings that AOSD patients with high IL-18 levels tend to present with systemic inflammation [29, 30]. Therefore, the levels of CLEC5A and IL-18 might be used to differentiate a systemic pattern from the chronic articular pattern of disease course.

Our longitudinal follow-ups of AOSD patients showed a significant decrease in the levels of circulating CLEC5A-expressing monocytes and granulocytes after 6 months of therapy, paralleling the clinical remission (Figure 5). The blockade of CLEC5A has been found to inhibit the activation of NLRP3-inflammasome and its downstream cytokine production [13]. In addition, we previously revealed that the anti-CLEC5A monoclonal antibody could inhibit Japanese encephalitis virus-induced proinflammatory cytokine release from microglia and prevent neuronal damage [31]. These observations indicate that CLEC5A may be targeted as a strategy for the treatment of infectious or inflammatory diseases.

Despite the novel findings presented herein, there are some limitations in our study. This was a preliminary, pilot study that enrolled a limited number of active AOSD patients. We also did not investigate the role of DAP10, another adaptor protein, in AOSD patients because of the implication of other different associated receptors, signaling kinases, and intracellular domains [32, 33]. Therefore, a long-term study enrolling a larger group of patients and including different ethnic populations is required for the validation of our findings.

## 5. Conclusion

Elevated levels of circulating CLEC5A-expressing monocytes and granulocytes, CLEC5A overexpression on PBMCs in our AOSD patients, and the positive correlation between CLEC5A levels and disease activity or inflammatory parameters of AOSD suggest that CLEC5A may be involved in AOSD pathogenesis and potentially serve as an activity indicator.

## Figures and Tables

**Table 1:** The correlations between CLEC5A expression and inflammatory parameters as well as proinflammatory cytokines in 34 patients with adult-onset Still's disease (AOSD).

CLEC5A expression	Frequency in monocyte (%)	MFI in monocyte	Frequency in granulocyte (%)	MFI in granulocyte
Activity score	0.400^∗^	0.438^∗^	0.388^∗^	0.506^∗∗^
Ferritin levels (*μ*g/l)	0.379^∗^	0.271	0.405^∗^	0.461^∗^
IL-1*β* levels (pg/ml)	0.361^∗^	0.284	0.033	0.245
IL-18 levels (pg/ml)	0.344	0.353	0.365^∗^	0.463^∗^

CLEC5A: C-type lectin domain family 5-member A; MFI: mean fluorescence index; IL-1*β*: interleukin-1*β*; IL-18: interleukin-18. ^∗^*p* < 0.05 and ^∗∗^*p* < 0.005 by the nonparametric Spearman's rank correlation test.

## Data Availability

All data relevant to the study are included in the article.

## Abbreviations

AOSD: Adult-onset Still's disease
CLEC5A: C-type lectin domain family 5-member A
ELISA: Enzyme-linked immunosorbent assay
FITC: Fluorescein isothiocyanate
HC: Healthy controls
IL: Interleukin
MFI: Mean fluorescence intensity
PBMCs: Peripheral mononuclear cells
PMA: Phorbol 12-myristate 13-acetate
PE: Hycoerythrin.
